# Smart Parking System with Dynamic Pricing, Edge-Cloud Computing and LoRa

**DOI:** 10.3390/s20174669

**Published:** 2020-08-19

**Authors:** Victor Kathan Sarker, Tuan Nguyen Gia, Imed Ben Dhaou, Tomi Westerlund

**Affiliations:** 1 Department of Future Technologies, University of Turku, 20500 Turku, Finland; tunggi@utu.fi (T.N.G.); imed.bendhaou@utu.fi (I.B.D.); tovewe@utu.fi (T.W.); 2 Department of Electrical Engineering, College of Engineering, Qassim University, Unaizah 56453-2865, Saudi Arabia; 3 Department of Technology, ISIMM, University of Monastir, Monastir 5000, Tunisia

**Keywords:** sensor node, architecture, IoT, LoRa, edge, cloud, energy-efficient, smart, parking, dynamic pricing, management, vehicle

## Abstract

A rapidly growing number of vehicles in recent years cause long traffic jams and difficulty in the management of traffic in cities. One of the most significant reasons for increased traffic jams on the road is random parking in unauthorized and non-permitted places. In addition, managing of available parking places cannot achieve the expected reduction in traffic congestion related problems due to mismanagement, lack of real-time parking guidance to the drivers, and general ignorance. As the number of roads, highways and related resources has not increased significantly, a rising need for a smart, dynamic and effective parking solution is observed. Accordingly, with the use of multiple sensors, appropriate communication network and advanced processing capabilities of edge and cloud computing, a smart parking system can help manage parking effectively and make it easier for the vehicle owners. In this paper, we propose a multi-layer architecture for smart parking system consisting of multi-parametric parking slot sensor nodes, latest long-range low-power wireless communication technology and Edge-Cloud computation. The proposed system enables dynamic management of parking for large areas while providing useful information to the drivers about available parking locations and related services through near real-time monitoring of vehicles. Furthermore, we propose a dynamic pricing algorithm to yield maximum possible revenue for the parking authority and optimum parking slot availability for the drivers.

## 1. Introduction

An increasing number of vehicles has become a problem lately and it is drawing significant concerns worldwide. Congestion of traffic is causing problems for the authorities in properly managing roads and highways to ensure timely trips; especially in developing countries, as the infrastructure is not as technologically advanced as in developed ones. Traffic jams are marked as critical limitations towards development [1]. In addition, cities and countries with large populations suffer even more as the scarcity of available roads and sideways are even worsened by improper and random parking in unexpected places. Furthermore, traffic jams due to lack of parking places and sporadic standing of vehicles on the road at unplanned areas causes significant stress and frustration [2]. This can trigger aggressive driving behavior to compensate for the lost time, which in turn, affects road safety [3]. For instance, studies reported in Norway [4] reveal that parking-linked accidents account for 2.4% of the total injuries in the country.

Drivers who want to lawfully park their vehicles are left in a helpless situation as they have to spend longer times to find an available parking place when visiting, often ending up in a late arrival or unnecessarily early departure if they want to reach their destination in time. Lack of information about available spots in advance causes delays in parking and sometimes it is impossible to find one. A surprisingly large number of working-hours is wasted due to traffic jams which hinders economic growth [5]. Moreover, while stuck in the traffic, the vehicles waiting on the road burn fuel unnecessarily resulting in an increase in greenhouse gases such as carbon-monoxide and carbon-di-oxide [6]. Smart parking is part of the solution in reducing air pollution and such systems play an indisputable role in the vision of smart cities worldwide [7].

The rise of Internet of Things (IoT) is a strong enabler for smart cities. IoT has facilitated real-time monitoring and control with the power of connectivity, information exchange and intelligent processing of data. In addition, through the evolution of IoT, advanced technologies such as machine learning, sensor fusion and real-time analytics are now integrated in it. In the case of the vehicle parking problem, an IoT-based system can play a significant role. By collecting real-time data about vehicles and parking slots, it is possible to build a smart system which manages the allocation of parking places, provides real-time monitoring data to the authorities and informs the drivers about availability of nearby parking places [8].

**Figure 1:**
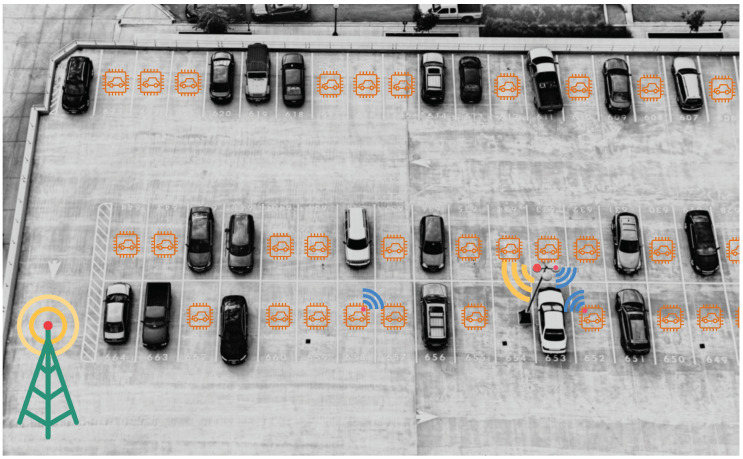
Use case of proposed system in a typical parking scenario. The nRF and LoRa communication are shown in blue and yellow, respectively.

Keeping in mind the aforementioned aspects, in this paper, we propose a smart solution for simplified, reliable and easily manageable vehicle parking system as depicted in Figure 1. We incorporate sensor nodes comprising of multiple sensors which allow us to detect vehicles and to acquire contextual and environmental information. In addition, we implement edge computation capable gateway. Edge computing brings Cloud-like computational resources closer to the source of data [9]. By harnessing the advantages of an Edge computing architecture, the network load is reduced to the minimum, lowering the required number of gateways that must be placed across a certain area. To create a reliable and secure network for the parking system, we use the nRF [10] and LoRa [11] wireless communication technologies which are optimal considering the amount of transferable data and the use of network bandwidth. These technologies also reduce the installation costs significantly, as compared to traditional approaches that use Wi-Fi of Bluetooth Low Energy (BLE) communication technology which require a much larger number of gateways. We provide a proof of concept with a specific realization to demonstrate the viability and usability of the proposed smart parking system. Furthermore, we present a novel algorithm for dynamically setting parking fees for maximum revenue by optimally balancing between available parking spots and parking requests while ensuring the minimum parking fee is always collected. We perform experiments both in a real-life parking area and simulate algorithm for evaluating the performance, analyze the results and discuss possible scopes of improvement. The main novelty is at a theoretical level providing the following:

- A layered architecture for smart parking system consisting of occupancy detection sensor nodes, LoRa communication and Edge-Cloud computing.
- Implementation of low-cost, energy-efficient and secure parking sensor nodes with real-time multi-parametric measurements.
- A novel algorithm for dynamically determining parking fee for maximum revenue.

The rest of the paper is organized as follows: Section 2 goes through existing related works on smart vehicle parking management systems and motivation for this work, Section 3 presents our proposed multi-layer architecture, Section 4 presents our dynamic pricing algorithm, discusses features and Edge services provided by our system, and points out design considerations. Section 5 exhibits the proof-of-concept primarily focusing on the lowest hierarchical levels, Section 6 explains the experimental setup and obtained results. Finally, Section 7 concludes this paper and provides direction towards future works.

## 2. Related Work and Motivation

Parking management is a common challenge when battling with the problem of traffic mismanagement and congestion. Several works have been performed to alleviate the difficulty in managing parking of vehicles.

P. Sadhukhan [12] implemented an IoT-based e-Parking system to address real-time detection of available parking slots using a meter which enables automatic collection of parking charges from the users. A camera is used to recognize a vehicle by reading the number plate and extracting the registration number. The meters connect via Wi-Fi to a nearest laptop acting as a local server. The laptop is connected to the Cloud where a central parking management software runs. The server sends short messages (SMS) to parking area operator and users about various parking related information through a serial port-based GSM module. Although the automatic system enables convenient parking for drivers and authorities; however, there are several limitations. The use of camera and Wi-Fi communication in each meter is expensive and causes high energy consumption. Besides, the micro-controller (MCU) interfacing the camera is inadequate for on-board image processing. Moreover, the authors did not present any fail-safe mode of operation when the system loses connectivity.

In another work, Aydin et al. [13] presented an IoT-based platform by using genetic optimization technique for smart cities based on navigation and reservation. A device consists of an MCU, a magnetic sensor, ZigBee communication module and a battery. It connects to a gateway which forwards real-time parking space availability data to the Internet via GPRS. While using a genetic algorithm in the Cloud to find nearest parking place is quite innovative, the system has severe shortcomings. For example, using only a magnetic sensor is not enough to accurately detect presence of a vehicle. The system does not authenticate vehicles for parking rights. Besides, as road lengths differ dramatically, placing one gateway per street would not provide full coverage. In some cases, it can result in inefficient placement of gateways, i.e., too many gateways in close proximity. Moreover, there is no inter-gateway information exchange without first reaching the Internet. Therefore, an interruption in the network could end up in data loss and error.

Managed parking systems often have automated parking fee collection and billing which allow time-based booking. Hainalkar et al. [14] presented a smart parking pre-and-post-reservation billing service. The system consists of a control unit and a local server unit. The control unit has an infrared (IR) sensor, radio frequency identification (RFID) reader and an LCD display. The first type of control unit uses a MCU while the other one employs Raspberry Pi as the controller. Connected through *Google* cloud service, another Raspberry Pi acts as a local server. The parking slot availability and its location data are accessible from an Android application. However, the system is not scalable as each local control unit and server uses one single-board computer making it costly when the number of the parking slots increases. Besides, IR sensor cannot reliably ensure presence of a valid vehicle. Furthermore, the system does not provide any monitoring service to the authorities.

Grodi et al. [15] proposed a wireless sensor network (WSN)-based parking space monitoring and visualization system. It has a 3-layer hierarchy: an XBee-based WSN among sensor nodes, a local Internet gateway, and a cloud database with a *Node.js*-based server. The authors provided a mobile application for viewing real-time parking occupancy. However, with no direct connection to the gateway, performance and response time can degrade in a large parking area due to multi-hop communication. Besides, use of ultra-sonic sensors is misleading, for example, a cat can trigger a false feed that a parking spot is occupied. Ramaswamy et al. [16] proposed a parking system with Raspberry Pi, camera and ultra-sonic sensor to reduce greenhouse emission. However, it is not feasible for large parking areas and the detection performance is limited. Similarly, Wang et al. [17] presented an optimal path guidance for the drivers for shorter access time to the nearest parking slot. However, use of one sensor type for detecting vehicle’s presence is insufficient. Besides, GSM connection is costly, requires maintenance and network coverage might not be available everywhere.

Kanteti et al. [18] presented a system for commercial areas in smart cities focusing on improved algorithm for parking slot management using Raspberry Pi, passive IR sensors, and IP cameras. It detects the vehicle registration number at the parking entrance and sends it to a database in the Cloud. Afterwards, billing starts and the user is notified by SMS. While it finds the nearest available parking slot, PIR sensor-based detection is deficient. Besides, camera-based authentication can induce legal problems and might be impractical due to weather conditions such as heavy snowfall or fog. In addition, large-scale deployment is costly due to use of camera and Raspberry Pi. Teller et al. [19] developed a framework named *SParkSys* which forms a camera sensor network for effectively recognizing the vehicles with image processing in Raspberry Pi. However, it is expensive, and a failure in the central node or a network connectivity problem can lead to a complete system stall.

Differences in usage, weather, authority, local regulations, number of users, and related services make it difficult to choose a specific type of sensor for detection. Besides, there are inherent challenges when implementing IoT-based systems in terms of manageability and deployment of sensor nodes [20]. Pham et al. [21] proposed an architecture to unify software services for parking management based on GS1 standard for prototypes deployed in different places. While it is scalable, the sampling rate of the sensor node is quite high for securely transferring real-time information to the authorities. Silar et al. [22] illustrated a similar system for large-scale deployment in smart cities. They analyzed variation in occupancy and claimed good system performance when multiple detectors are placed on road-side parking places. However, the system suffers from high-latency sampling when sending data to server, i.e., the system registers vacancy long after a vehicle has left the parking slot. A detailed survey by Hassounen et al. [23] investigated several parking systems and detailed on the sensor and communication technologies used. In particular, they observed advantages such as higher availability, cost reduction, efficient billing, reduced traffic congestion, fast system response, helpful services and effective management. For vehicle detection, mostly IR sensors, ultrasonic sensors, RFID tags and simple light sensors are used. More expensive systems use IP-based and CMOS cameras for authenticating and validating vehicles, especially at the entrance and exits of a confined parking area. MCU-based sensor nodes are common; however, Raspberry Pi and similar single board computers are used for image processing-based authorization. Wi-Fi and ZigBee communication are mostly used for networking among the sensor nodes and intermediate gateways. In contrast, GSM-based communication is preferred when sending data to Cloud from individual sensor nodes and the gateways.

While the existing systems use different methods and sensing techniques, their limited functionality cannot meet today’s parking needs. Table 1 enlists features and compares aforementioned works with our proposed system. Implementing multi-parametric sensor nodes in a parking system ensures higher accuracy of occupancy, real-time information and an opportunity for vehicle classification. While making the system less expensive, it also greatly reduces computational and energy requirements than when using a camera. This is crucial, especially in developing countries where cost-effective and comprehensive traffic management is preferred. Such solutions aid to decrease greenhouse emissions caused by unnecessary extra driving while searching for a parking space.

By avoiding a single gateway-centered scheme, we can achieve higher fault-tolerance and yield a more distributed system. In case of failure, another one can take over since all the edge gateways are synchronized. Existing systems collect and send data directly to the Internet which can result in data loss during a network interruption. By using reasonable buffer in the edge gateways, this is prevented and the stored data is transferred to the Cloud once network is re-established. Since edge gateways are architecturally situated near the sensor nodes, we harness Cloud-like capabilities while reducing the data flow across the network and limitations of mobile-Cloud computing [24]. For connecting the sensor nodes to the edge gateways, nRF is preferred over other short-range communication technologies such as ZigBee since nRF has low connection complexity, requires less power and achieves a high data rate. In contrast, LoRa provides long-range, inexpensive and low-power communication. For example, SigFox requires a licensing fee for use, Narrowband IoT (NB-IoT) depends on 4G LTE coverage which is not available everywhere, and GSM modules are expensive for large scale deployment [25]. This makes LoRa suitable for inter-gateway network as gateways are placed far away and require less data to be transferred compared to communication between sensor nodes and gateways. Keeping the technological aspects of the sensor nodes, communication mediums, energy efficiency, expense, available features and limitations of the aforementioned systems, there exists a scope for developing a smart parking system. With our proposed system, we either eliminate or lessen the existing shortcomings.

## 3. Smart Parking System Architecture

**Figure 2:**
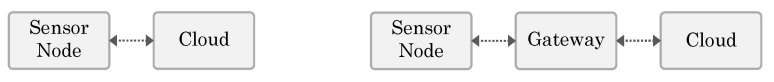
Typical system architecture in an IoT-based parking system.

An IoT-based parking system can be structured in multiple hierarchical levels, each of which serves a specific purpose. Typically, such a system senses the environment and/or actuates at the lowest level of the hierarchy. Shown in Figure 2, the sensor node is usually directly connected to the Cloud for data transfer, or, is connected to a nearby local gateway or server which enables data pass-through to the Internet and finally to applications located at the Cloud. A fast communication from the nodes to all the way up to the Cloud can result in a near real-time application experience. This is observed in recent years [23] and it works well for single node setup. However, as the number of nodes increases and ubiquitous sensor data becomes essential for IoT-based parking systems, the performance of this approach starts to deteriorate. Correspondingly, the reliability of the system can diminish and cost of maintenance may increase, especially in application scenarios in which real-time monitoring and control are of utmost importance.

**Figure 3:**
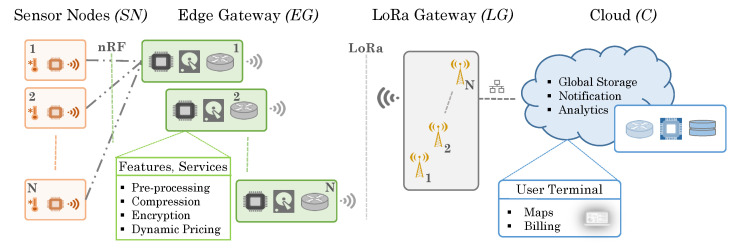
Proposed multi-layer system architecture.

To ensure real-time parking space monitoring and control, the existing and most commonly used Sensor-node-to-Cloud hierarchical approach needs to be improved by integrating high computation-capable computers or gateways working at the edge layer in the existing system. This would improve the overall performance and manageability, while effectively reducing operational latency and cost. In addition, by using LoRa communication technology where data is not required to transfer frequently and in high volumes, the cost of network coverage and infrastructure can be significantly lowered. Figure 3 illustrates our proposed system architecture that can be used to implement the typical parking scenario depicted in Figure 1. Our proposed system consists of four layers: a sensor node layer *(SN)*, an edge gateway layer *(EG)*, a LoRa gateway layer *(LG)* and a cloud layer *(C)*. These layers and their corresponding functionalities are described as follows.

### 3.1. Sensor Nodes

At the lowest and outer limits of the proposed system are sensor nodes which are responsible for monitoring the parking area. A sensor node has multiple sensors to detect a vehicle and gather other environmental data. Such nodes are placed either underground in a car park or at the ceiling in a multistorey car park to detect parked vehicles using one or multiple sensors. For example, an infrared light sensor in combination with an ultra-sonic rangefinder can be used to detect the presence of a vehicle. However, car parks are exposed to different weather conditions, and therefore a magnetometer to detect the specific variation in the magnetic field or a thermal camera to detect differences in temperature should also be considered. In more sophisticated and privacy-invasive parking systems [26], it is possible to clearly identify vehicles using visual sensors, e.g., RGB cameras. However, this is expensive, and requires higher processing power and specific placement, thus limiting their usage to particular scenarios.

In most cases, sensor nodes are run by batteries, and hence are quite limited in terms of resources such as processing capability and communication methods. Therefore, the acquired data is sent to a nearby edge gateway for further processing before it can be used at higher level applications. Owing to the battery-run implementation, it is important to select an appropriate energy-efficient communication technology to help extend the run-time of the parking sensor nodes. Two possible low-power communication technologies to connect sensor nodes to edge gateways are Bluetooth Low-Energy (BLE) and Nordic Semiconductor’s nRF, of which nRF has the highest operating range.

### 3.2. Edge Gateway

Edge computing enables relocation of some of the processing and analysis out of the Cloud close to the lowest hierarchical layer where the data originates; edge computing brings the cloud paradigm to the edge of the network [9]. This provides a robust solution for IoT-based systems which require computation-intensive applications and services to fulfil the requirements and comply with standards. Edge gateways are powerful computers whose resources are not limited by battery capacity when compared to sensor nodes. Therefore, data pre-processing, filtering, compression, and encryption can be done in edge gateways as these processes are relatively complex and require higher computational power. With powerful computing, we can build a more robust system to tolerate sporadic or intermittent network connectivity issues by implementing fallback algorithms to ensure operation until network connection is re-established. Higher computation power also allows task sharing among the high-speed network-equipped gateways if one of the gateways has too much execution load. Furthermore, to optimally distribute processing in edge gateways, a cloudlet and greedy auction-based computation can be implemented among the edge gateways [27].

The edge layer consists of multiple edge gateways, each of which can simultaneously handle multiple sensor nodes. If the number of sensor nodes exceeds the maximum allowed simultaneous connected devices, a collaborative task allocation and handover of sensor nodes can be implemented to share the computational load among multiple edge computers for reducing overall operational latency. Normally, sensor nodes are connected to the nearest edge gateway, but they can switch to another edge gateway if there is a connectivity problem, thus increasing the robustness of the system.

### 3.3. LoRa Gateway

To cover a wide area such as urban, suburban and even rural areas at the time of events, we propose a new LoRa gateway layer. As its name implies, this layer connects to the edge layer using the LoRa communication technology that can cover a very wide communication range up to several square kilometers of area [28]. To increase the range of a LoRa network, it is possible to deploy its mesh network capability; one of the LoRa gateways is connected to a high-speed Internet connection to transfer data to a cloud server. The LoRa gateway layer can be bypassed if there is no need for long-range communication and an edge gateway can be directly connected to high-speed Internet. This is possible because there is no data processing located in the LoRa layer. It does provide, however, unbeatable benefits in terms of communication range and cost effectiveness. Owing to its economic benefits, the LoRa gateway layer is used to increase robustness of the whole system by providing an alternative access point to a network.

The LoRa specification has some specific guidelines for the transmission and use of the frequency spectrum. A proper carrier frequency along with other LoRa configuration parameters according to the local regulation should be chosen which is robust and provides reliable quality of service (QoS) [29,30]. It should be also noted that LoRa has a maximum transmission power limit of 25 mW and a maximum duty cycle of 1%. Furthermore, a typical LoRa transceiver chipset is comparatively cheaper than other similar wireless communication modules and has a small physical footprint. These make LoRa communication suitable for IoT-based applications where it is not required to continuously send a large amount of data and when long-range operation and high energy-efficiency is desired [31]. Since LoRa is an open access medium, other devices can access the same frequency spectrum. Therefore, it is essential that security is inbuilt into the system throughout the whole architecture starting from the sensor nodes [32] and that LoRa gateways can distinguish, validate and authorize the edge gateways which connect to it. Accordingly, edge gateways must use strong encryption and data validation when sending data to a LoRa gateway. Furthermore, to ensure appropriate data access and restriction of non-permitted operation, a suitable authentication scheme [33] should be employed.

### 3.4. Cloud

The cloud layer is the topmost layer in the architecture and visible to the end-user by providing different services including virtually unlimited storage and computational resources spread across the globe. From our smart parking system point of view, the cloud layer enables features such as usage analytics, revenue monitoring, automatic payment management and other related services for enhancing user experience. Applications running in the web or installed in handheld smart devices such as smartphones can provide real-time information and directions to nearby parking areas. Besides, reservations can be securely made online for pay-as-you-go or on a monthly basis which saves time and hassle. Anonymous payment schemes and driver information hiding can be employed to further increase monetary transaction security and enhance location privacy of the drivers [34].

In the long run, Cloud-accumulated parking data can be used to develop smarter algorithms to improve user experience. Authorities and involved industries can use this data to understand the needs of the users, parking usage pattern and forecast parking trend, and accordingly design the system to make the management more efficient [35]. This kind of big data is supremely useful for different alliances among the authorities from multiple countries, companies and organizations which work on standardizing parking, traffic management, and improvement of smart city solutions and to reap the most possible benefits [36]. Automated analysis running across numerous servers in the Cloud can enhance the system without too much human intervention. The Cloud also enables services such as real-time situational feedback and push notifications for end users and case-specific emergency alerts to the authorities. For example, along with other sensors, visual data from cameras connected to the Edge and mounted on special places such as at the entrance and exits of a parking area can provide highly useful information in situations such as when a parking rule violation or an accident occurs.

## 4. Features, Services and Considerations

A typical parking system becomes smart when it can sense vehicles, and collect, analyze and use data for adaptive decision making resulting in a reliable and convenient user experience. In this section, we discuss features, services and design considerations of our proposed system.

### 4.1. Dynamic Pricing

One of the most important aspects of a parking system is how much a customer or a vehicle owner has to pay for using the parking space and how the fee is determined. If managed appropriately, this has twofold advantage- one for the parking customers and the other for the parking management authorities. Depending on the demand of the parking spaces, especially during peak hours of the day, the authority can set a slightly higher price to keep a balance between the demand and available parking slots while offering customers a lower parking charge when more slots are available. For example, such a scheme can be very fruitful in a large shopping mall parking where there are limited parking slots but lots of cars and drivers are required to be guided to the nearest parking slot. Furthermore, it can help prioritize parking requests based on importance, time of request and special requirements (e.g., drivers with some disabilities).

Dynamically pricing the parking slots is a relatively recent concept; however, it has already started gaining popularity in many places in the world such as in Washington DC [37]. Parking management system aims at maximizing the revenue for the operating company. For the user side, the system helps to reduce the fuel consumed for commuting from the current location to the parking slot. The work reported in [38] defined functions which affect the cost of a parking: driving, waiting, parking and walking. The optimization problem is solved using the greedy parking slot allocation algorithm. Parking management using dynamic pricing arises due to two objectives. The first one is to match supply and demand. In case the number of vehicles requesting parking services in one area exceeds the number of available parking slots, the price should be increased such that only commuters with an urgent need can access the parking. The second objective is to reduce the traffic congestion by reducing the parking price in less congested area. To fulfil these two objectives, we propose a novel algorithm which dynamically assigns the hourly price for each parking area.

Let *M* be the number of parking slots and *N* be the number of vehicles requesting the parking. Our goal is to maximize *f* where $P_{j}$ is the hourly price of the *j*th parking lot.

(1) $$f=\sum_{j=1}^{M}P_{j}$$

When the number of vehicles exceeds the number of slots in a particular parking area of the city, the parking management companies seek to maximize the objective function (1). This can be expressed as (2) where $N_{opt}$ is the number of users who can afford the current cost of parking, and $U_{j}$ is the maximum cost that the *j*th driver can afford. The bidding is enabled when there are fewer available slots *M* than the number of drivers $N_{opt}$. From (2), for all parking slots (indexed with *j*), until up to *M* number of available slots, it prioritizes the highest ones from the list of drivers when the bid value is more than the current set price of an individual slot. In the case when a driver fails to win the bid, the system will put them in the next nearest parking area request queue where there is comparatively less demand. This happens in real time, as it is processed at the time of the request is made. The parking system’s algorithm running in edge and cloud gateways will perform this in a synchronized manner so that drivers do not have to bid multiple times, nor does it become a hassle.

(2) maxfsuchthatM≥NoptUj≥Pj;∀j≤M

During an hour, a parking area with 12 slots gets online parking requests from 20 drivers. Based on needs, drivers also notify the maximum price that they can afford. To enforce fairness of usage, an upper bound of bidding is set by the parking authority so that a parking request with an unfairly high bid is not allowed. Consequently, a pricing scheme is selected so that the revenue is maximized. The algorithm starts by assigning the maximum value and then gradually reduces the price so as to maximize the revenue and match demands with supply. For example, if 2 drivers bid 15€, 3 drivers bid 10€ and 7 drivers bid 3€, Table 2 shows three different pricing schemes, from which the price of 10€ is clearly the optimal one.

The pseudo-code for computing the dynamic price is described in Algorithm 1. The algorithm gets all *N* bids from the drivers and sorts them in a descending order. For each bid, it determines the number of drivers who can afford the bid and records as $N_{opt}$. Afterwards, the objective function is evaluated and the optimal value is then taken as the maximum value stored in the vector $f_{i}$. If the number of $N_{opt}$ is larger than *M*, the algorithm prioritizes the users based on time-stamp or descending order of bids for parking (not shown in the pseudo-code). The complexity of the algorithm depends on the complexity of the sorting algorithm. Merge sort or heap sort [39] is used for achieving a linear run-time (Nlog(N)) for searching maximum value using *N* number of comparisons, thus significantly reducing the overall processing time.
**Algorithm 1** Dynamic pricing.1:**procedure**dynamic pricing(*M*, *N*, $P_{bid}$)2:    Discard unfair bids from $P_{bid}$3:    Sort $P_{bid}$4:    **for** i←1,N **do**5:        $N_{opt}\leftarrow$ number of drivers who can afford $P_{bid,i}$6:        $M_{i}\leftarrow min\left(M,N_{opt}\right)$7:        $f_{i}\leftarrow M_{i}P_{bid,i}$8:    **end for**9:    $f_{opt}\leftarrow max\left(f\right)$10:    **return** $\left(f_{opt},P_{opt}\right)$11:**end procedure**

### 4.2. Edge Services

Staying in close proximity of the sensor nodes, the acquired data from the nodes is sent as it generates, and is received at the Edge. After receiving, the data is checked for errors and recovery process is run if required. Following the pre-processing and summarization of data, to maintain integrity while reducing the overall packet size, the gateways compress data using a lossless algorithm before sending over the network. Unlike lossy variants, a lossless compression cannot achieve high compression ratio which should be taken into consideration when determining the maximum allowable nodes connecting to a single gateway [40]. At the Edge, intelligent algorithms employ greedy planning of data-flow reducing the total path which data travels resulting in an increased performance and lower response time of the system. In addition, it offers better reliability due to redundancy and smarter task reallocation in case of a hardware failure.

Multiple services run at the edge gateway layer for analyzing and extracting authority and customer-specific features and information from the raw data. In the next step, access control service validates or rejects the use of parking system depending on the status of the customer. If the edge gateway does not have previous information, it sends a request to the LoRa gateway for authentication. Being connected to the cloud database through high speed Internet connection, the LoRa gateways collect the information and then pass it back to the parking control system at the Edge. The dynamic price computation service based on Algorithm 1 then sets a total payable fee for the parking request and awaits confirmation. Consequently, the real-time map service in the cloud layer provides direction to the vehicles, significantly easing and shortening the time to reach the parking space otherwise. When the parking slot is emptied, the billing and payment service charges the fee from the customer, if not paid earlier due to uncertain parking duration.

Specific automated data and system maintenance routines are periodically run in the edge gateways to accumulate information on hardware status of parking sensor nodes. Monitoring of network connectivity parameters, proper bandwidth and channel allocation, availability of a node and fallback configuration services enhance the quality of service (QoS). For instance, if there is a loss of connectivity, the edge gateway layer continues to perform most of the operations and when network communication is re-established, it updates the cloud layer accordingly. Although not realized in this work, another possibility to use aggregated system data is to create digital-twin-based predictive maintenance; detailed real-time updates on hardware status criterion such as number of general run-time faults, number of critical errors, battery health and board temperature can be monitored by running a background service at idle times with a lower priority. Predictive maintenance is essential to reduce the operational and maintenance cost over prolonged periods of time. Altogether, the services running at the Edge ensure that the parking management is simplified and costs are reduced while having optimum revenue, and also leave room for easily adding more services in future.

### 4.3. Considerations

We used the nRF communication technology for connecting the nodes to the nearby edge gateway. It serves well in terms of communication range and energy-efficiency. However, when the number of connected sensor nodes increases in a parking area, a single gateway solution is not suitable anymore. One limiting factor for the number of sensor nodes is the nRF communication technology based on nRF24L01+ [10] which can handle a maximum of 126 channels. If required, two or more nRF modules can be used in a single edge gateway to accommodate more nodes per edge gateway. Alternatively, using more than one edge gateway reduces the number of connected sensor nodes and ensures better signal strength between communicating parties. This is preferred because there might be other 2.4 GHz frequency band-based devices, such as cordless telephones and Wi-Fi routers in the vicinity. Therefore, it is recommended to keep the number of connected sensor nodes well below the maximum possible to avoid data corruption due to interference. Besides, depending on the minimum accuracy and data rate of the sensors which are required in the parking application, other technologies such as the latest Ultra-wideband (UWB) communication can also be used.

The use of the LoRa communication technology provides a cost-effective, low bandwidth solution for connecting edge gateways to a LoRa gateway. However, some important aspects must be ensured for proper operation in the long run. In particular, the airtime of the transceivers must follow the local regulations and recommended transmission guidelines set by the law and telecommunication authority. When trying to achieve closer real-time-like behavior with LoRa, it is not possible to transfer unprocessed raw data directly to the Cloud through the LoRa gateway. Compression and data fusion are not the only techniques to target real-time-like behavior but also a sub-band frequency and channel hopping technique [41] can be applied. In the sub-band frequency and channel hopping, the communicating parties jump from one carrier frequency to another within the permitted frequency spectrum. This can increase the total realized data rate without violating the LoRa standard regulation of maximum duty cycle of 1% at a specific carrier frequency.

## 5. System Implementation

We provide a proof of concept with a specific realization to demonstrate the viability and usability of the proposed smart parking system. We mainly focus on the sensor node and the overall architecture. We implemented multiple similarly configured edge gateways and a LoRa gateway to test transmission of data from parking sensor node all the way to the cloud layer. Specific implementation details are described in respective sections as follows.

### 5.1. Sensor Node

A sensor node has a control unit and multiple sensors for collecting data in the parking area. For our proof-of-concept experiment, a Pico-power series AVR MCU from *Atmel* is used as the sensor node’s controller to minimize power consumption. For sensing, we used a 9-DOF (degree of freedom) MPU9250 [42] inertial measurement unit (IMU) from *Invensense* and a BME280 [43] environmental sensor from *Bosch*. The MPU9250 is used for detecting acceleration due to vibration when the engine of a vehicle is running during parking. Additionally, the magnetometer inside the MPU9250 measures the variation in magnetic field as a result of large amount of metal in the chassis of the vehicle. The BME280 measures temperature, humidity and barometric pressure for understanding the weather conditions of the parking area. Based on the sensor readings, the sensor node achieves context awareness and reports it to one of the edge gateways. Furthermore, connected to an internal ADC of the MCU, a sensor placed on the outer surface of the node detects any water that may have accumulated in the parking area. This enables identifying flooded slots and reporting to the authority for appropriate maintenance action, and to the drivers for avoiding those slots.

#### 5.1.1. Connectivity

**Figure 4:**
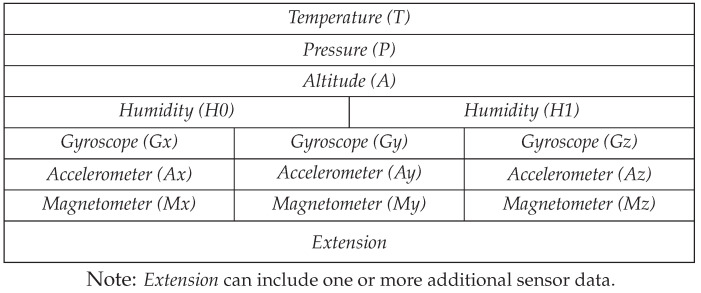
Sensor node to edge gateway data transmission packet format.

For connecting to the nearby edge gateway, an nRF24L01+ based communication module from *Nordic Semiconductor* [10] is used which allows simultaneously connecting several parking sensor nodes to the same gateway. Shown in Figure 4, a specific packet structure is defined for reliable and identifiable data transmission from sensor nodes to the edge gateway. The nRF communication protocol provides 126 channels in the range of 2.4–2.525 GHz. Each module can use 6 logical data-pipes for receiving simultaneous individual data streams from other nRF transmitters. This enables multi-node connectivity to a single edge gateway. To reduce total transferable data from a sensor node to an edge gateway, a dynamic packet consisting of only required data parameters is used. Furthermore, least significant bits (LSB) of some data parameters such as acceleration and magnetic field are safely ignored as those fluctuate randomly. The data collection from a sensor node is switchable between one shot, streaming and on-demand streaming mode. In idle state, i.e., no vehicles are introduced to the parking slot, a periodic one-shot data is sufficient. When an approaching vehicle is detected, the sensor node can collect a single-set of data or stream data continuously for a certain period to get up-to-date and detailed information.

#### 5.1.2. Energy-Efficiency

**Figure 5:**
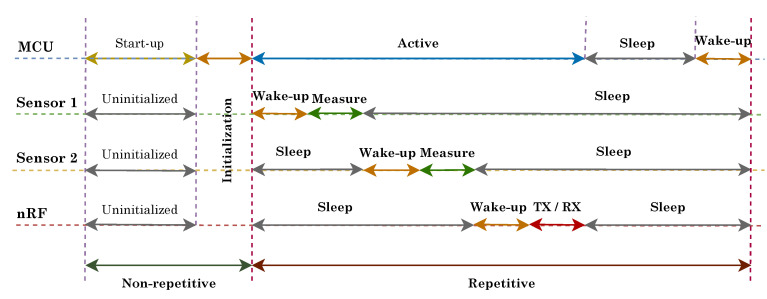
Operational time-line of the parking sensor node (Image not scaled to time).

To save energy, a greedy approach is applied in the sensor node to maximize the time in sleep mode and complete other operations such as measurement and data processing within shortest possible time. The operational time-line is shown in Figure 5. Essentially, the time-line is divided into two parts: a one-time non-recurring part which consists of start-up routines and initialization of sensors and communication module, and a recurring one which contains measurement, data processing, data transmission and sleep operation. The sleep duration in the latter part depends on the frequency (e.g., once every 10 s) at which the parking slot needs to be monitored for a parked vehicle. To achieve maximum battery life, all of the components including the MCU are kept in sleep mode for most of the time during operation and are only activated for the duration when required.

#### 5.1.3. Data Security

**Figure 6:**
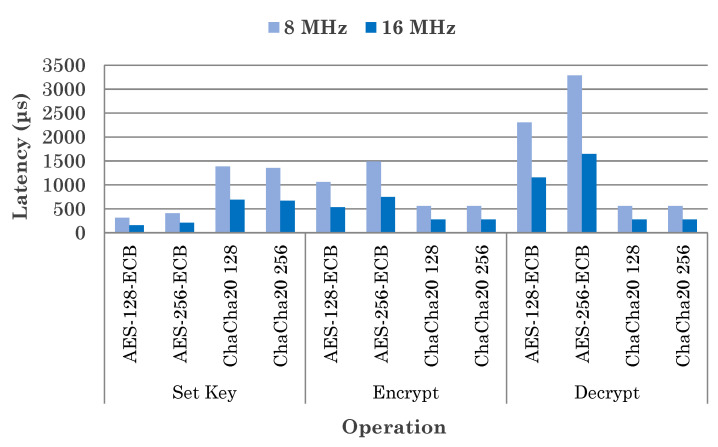
Comparison of latency in different operations of AES and ChaCha cryptography algorithms.

To secure the collected data from these sensor nodes before sending it upwards to an edge gateway, a suitable encryption is applied based on the operation mode. For instance, the AES [44] block cipher is used when only a single data collection is performed, while ChaCha [45] stream cipher is better when streaming continuously. As ChaCha is optimized for streaming data and has lower latency than AES, it is more suitable for the limited-resource MCU used sensor nodes. We used the Arduino *Crypto* library [46] to implement these cryptography algorithms in our sensor node. Figure 6 shows comparison of the operational latency in different operations of encryption process between the two algorithms when varying key sizes at different MCU clock frequency. Consequently, this approach also reduces energy consumption and helps to achieve longer sensor node battery life.

### 5.2. Edge Gateway

An edge gateway is a significantly more powerful device than a sensor node or the LoRa gateway. We used a Raspberry Pi3 B+ (hereafter referred to as Pi3) [47] running Linux operating system as the computing resource. The edge gateway provides services such as authentication, data encryption/decryption and data compression. For connecting the sensor nodes to Pi3, an nRF communication module with external antenna of 2 dB gain is used to ensure proper signal strength when a parking slot is occupied. For connecting to a LoRa gateway, a *Dragino* LoRa GPS HAT shield version 1.0 [48] containing an SX1276-based LoRa module is used which operates at 868 MHz carrier frequency. For better signal reception with LoRa, an external antenna is used. The *Dragino* LoRa shield also contains a GPS module; however, it is left unused. The GPS module can be used, for example, to provide localization of the gateways during setup and maintenance of the system. The gateway along with the communication modules is powered from a wall-adapter which runs at 220 V AC and provides the system a regulated output of 5 V DC at 4 A supply current.

**Figure 7:**
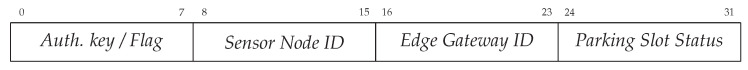
Edge gateway to LoRa gateway data transmission packet payload format.

As the edge gateway analyzes, processes, and extracts required information from the raw data collected from the sensor nodes, an encrypted summarized status of the parking slots is sent to a LoRa gateway. A simplified data packet payload structure comprising of 4 bytes is shown in Figure 7. For example, if 50 sensor nodes are connected to an edge gateway, 200 bytes of total data can be transferred to a LoRa gateway once every 31 s with 125 kHz bandwidth, spreading factor 7, a preamble of 8 bytes, coding rate of 1.25 and CRC checks. However, due to the implemented compression, the actual number of transferable bytes is lower than the calculated one.

We used the LZW lossless compression method [49] when transferring data from the edge gateway to the LoRa gateway. With LZW, we have the least possible amount of data transmitted while preserving data integrity helping to comply with the LoRa airtime regulation (i.e., maximum 1% duty cycle) by reducing the data size. Table 3 shows the compression and decompression latency for different number of connected nodes. Two different sets of data- a real-life sensor node data set and another non-repetitive true-random data set are used in the test to understand the normal and worst case results, respectively. Also, the test was run for 1000 times to get a true-to-reality average as Pi3 also runs other services and results can vary by few micro-seconds depending on the instantaneous processing load. It can be observed that despite being a lossless compression method with low compression ratio, as the number of parking sensor nodes increases, the real data achieves higher ratio and lower average latency compared to the theoretically generated worst-case true-random data.

### 5.3. LoRa Gateway and Cloud

The emphasis of this work is at the lower hierarchical levels, especially at the sensor nodes and edge layer. The LoRa gateway and cloud layers are abstracted more to cover the whole data flow from a sensor node to an end user. A LoRa gateway in the experiment connects to all the edge gateways for receiving extracted, summarized and processed data. To simplify the realization, another Pi3 is used as the LoRa gateway with similar configuration as of the edge gateway. However, this Pi3 is connected to a local 100 Mbps bandwidth Internet connection via the on-board RJ-45 Ethernet port.

At the cloud layer, we used a *Linode* [50] Cloud-based control and information display panel similar to [51] which runs parking information application and real-time notification service. The Cloud server aggregates all the data from different LoRa gateways spread around over a large geographic area. It provides powerful computing, storage capacity as needed and ability to run applications and services required by the system. For security, it employs advanced OpenSSH features, secure file transfer protocol (SFTP), *dm-crypt*-based file encryption and log-parsing application for detection of automated attacks to the server [50,52]. In this work, our Cloud implementation enables us to view the near real-time information of the parking slots.

## 6. Experimental Results

We ran the implemented smart parking system to assess its performance regarding energy-efficiency, data acquisition and vehicle-type sensing capability of the sensor node. We also tested our dynamic pricing algorithm to demonstrate its effectiveness for increased revenue.

### 6.1. Energy-Efficiency

**Figure 8:**
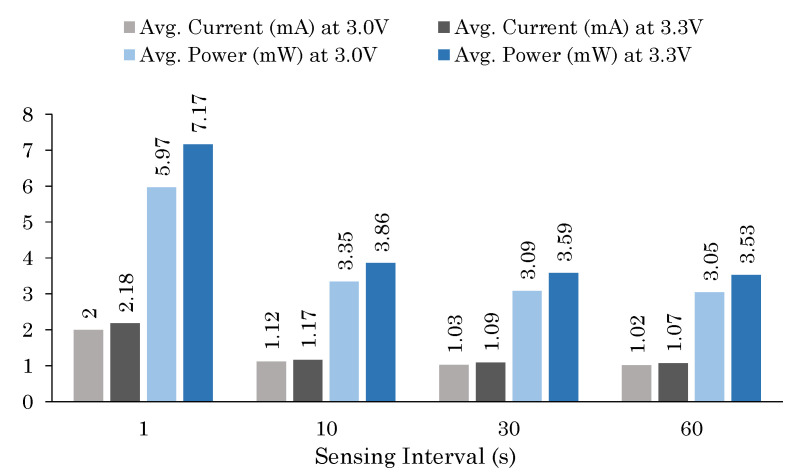
Average current (mA) and average power (mW) consumed by the prototype parking sensor node at different voltage levels and sampling intervals.

**Figure 9:**
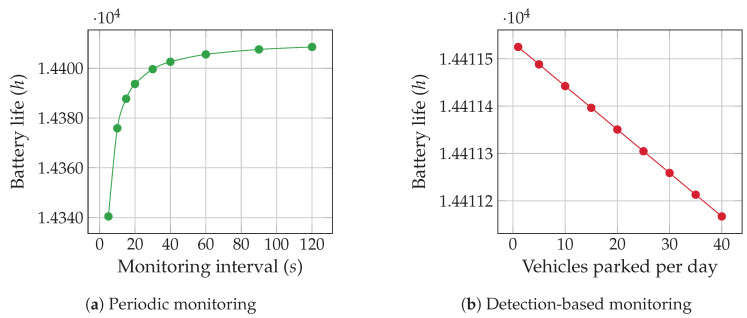
Sensor node’s battery (10,000 mAh) life during Periodic and Detection-based monitoring.

The energy consumption of our sensor node with different sensing intervals is shown in Figure 8. The measurements show average current and average power consumed when 3.0 V and 3.3 V voltage supply was used as the power supply. It can be observed that the overall energy requirement of the prototype sensor node is low because both the MCU and sensor nodes use low-power sleep mode. Among the test configurations, the lowest average current and average power are achieved at 3.0 V when vehicle detection is performed once every minute. This translates to an operating life of more than 1 year and 8 months with a 10,000 mAh battery when it works 24/7. However, if we switch to vehicle-detection-based transmission mode, lower number of transmissions occurs, and thus we can achieve a higher battery life compared to periodic vehicle monitoring mode, especially during the off-peak hours. Figure 9 shows how battery life is affected when a sensor node is run in these two modes: Figure 9a shows battery life for monitoring interval in seconds and Figure 9b for varying number of vehicles parked in a day. It is noticeable that in the periodic monitoring mode, the battery life can have higher variation depending on the monitoring interval. When calculating the battery life of the parking sensor node, a nominal 1% monthly battery self-discharge rate is used. Since the parking sensor node is mostly kept in sleep mode, the battery has enough time to recover the capacity lost due to switching from sleep to active mode [53].

For the experiment, the sensor node was composed of off-the-shelf modules and sensor boards. As these boards have extra components such as voltage regulators and other passive components, the sleep mode current of 0.69 mA at 3.0 V supply is undoubtedly high. This can be reduced by designing a single board PCB, hence eliminating redundant components and shortening wire connection lengths. During multi-voltage testing, it was found that a sensor node can also reliably operate at 2.8 V supply with brown-out detection enabled at 2.7 V. If the supply level is ensured with appropriate regulation, this can be lowered until 2.4 V without hindering the normal functionality of the node and the brown-out detection feature can be safely disabled. The battery life can be further optimized to prolong the battery life by aggressive use of advanced sleep modes, using minimal hardware components, applying selective data acquisition from sensors, implementing true shutdown technique, and, furthermore, with a fabricated System-On-Chip (SoC).

### 6.2. Sensing

**Figure 10:**
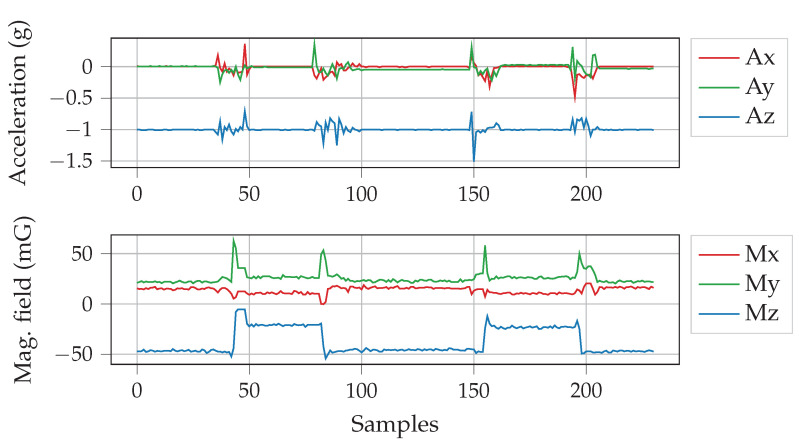
Variation in accelerometer and magnetometer readings sensed by our prototype parking sensor node when a vehicle enters and leaves the parking slot. Here, Ax, Ay, Az indicate 3-axes acceleration changes, and Mx, My, Mz indicate 3-axes magnetic field changes.

**Figure 11:**
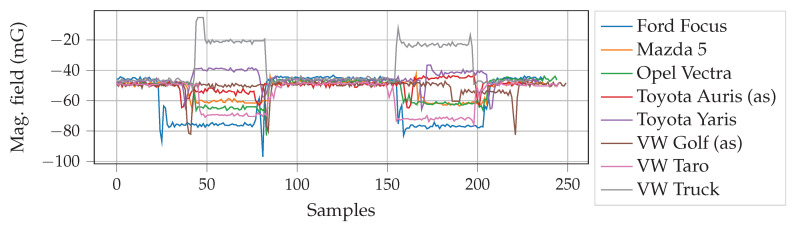
Variation in magnetic field (Z-axis) among vehicles when entering and leaving the parking.

**Figure 12:**
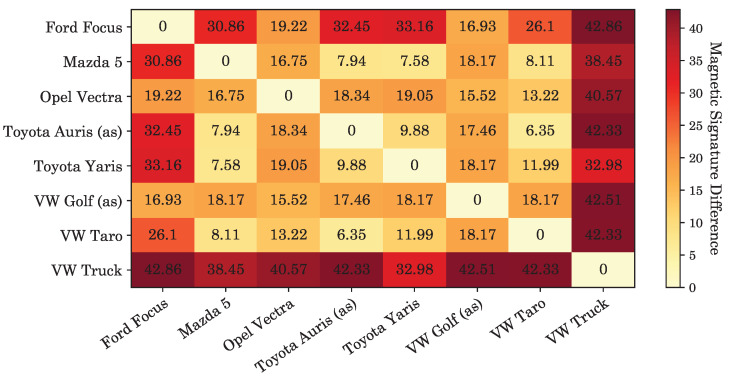
Difference in magnetic field signature (Z-axis) among vehicles.

To sense the change in magnetic field and the level of vibration when an empty parking slot is taken by a vehicle, the sensor node was placed on the ground keeping the sensor side near the surface. The sensor node clearly senses when a vehicle enters and leaves the parking slot; the readings of the acceleration and magnetic field changes are shown in Figure 10. Each of the parameters accurately registers a vehicle occupying a slot, but fusing the data increases the robustness of the system. Figure 11 shows the acquired data at a sampling rate of 4 Hz. During the test, the vehicles were parked and removed twice, subsequently. For presence detection, the data is filtered using a simple sample-and-hold transient filter, i.e., any sudden change shorter than a preset time period is ignored. Afterwards, the magnetometer data-points are cross-validated with acceleration data-points. For example, when there is a change in magnetometer readings, there is also a simultaneous fluctuation in the acceleration readings. This change happens due to deceleration and acceleration when a vehicle gets in and out of the parking slot, respectively, and engine vibrations. It is observable that different kind and make of vehicles result in different magnetic field disturbance. This is used to determine vehicle types, i.e., a large covered van or a small car. For example, the VW Truck causes higher degree of change in magnetic field, as shown in Figure 11, compared to other vehicles tested. Figure 12 lists the difference in Z-axis magnetic signatures calculated from the collected data using Fréchet distance [54] at the edge gateway. Evidently, the VW Truck has the highest magnetic signature difference compared to other vehicles, as also indicated earlier in Figure 11. This vehicle classification data can be used in the proposed dynamic pricing algorithm to set an additional fee for commercially used vehicles. Furthermore, since parking behavior (e.g., speed, duration etc.) differs, to compensate the time-wise variation, dynamic time warping [55] can be used to align sensor data across different vehicles.

**Figure 13:**
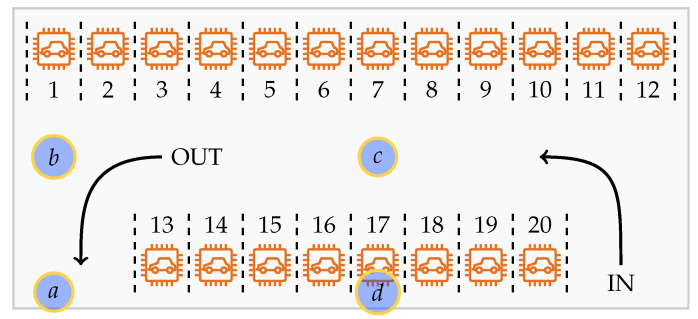
Edge gateway placement suitability in a parking slot during experiment. Gateway placement options are marked with *a–d*, and parking slots are marked with numbers 1–20.

The edge gateway is physically placed in a suitable place in the parking area such that the variation among distances from individual sensor nodes and the gateway is minimum. In other words, to ensure the best signal reception, the gateway should be at a uniform distance from each sensor node as possible. In our 45 m by 20 m experiment parking area, we tried four placement options for the edge gateway as shown in Figure 13. The edge gateway works well for all the placement options *a*, *b*, *c* and *d*, with option *a* getting the lowest connection signal. The reason is a large metallic dumpster (not visible in the figure) nearby slot 13 interfering the signal propagation when the gateway is placed at 1 m height from the ground. Placing the gateway higher with antennas kept upright improved signal reception, which should be considered during a permanent installation of the parking system.

### 6.3. Dynamic Pricing

**Figure 14:**
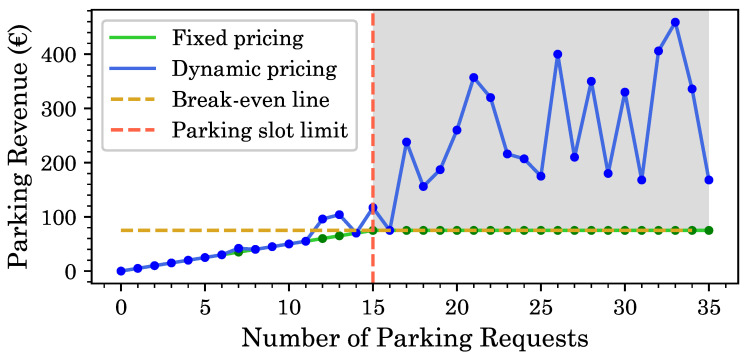
Comparison of fixed and dynamic parking fee scheme.

As shown in Figure 14, we simulated parking requests from drivers for a total of 15 parking slots and performed a comparison of dynamic and fixed pricing modes as the number of parking requests increases. In particular, when the number of requests is greater than the number of available parking slots (shaded in Gray), the dynamic pricing scheme increases revenue significantly. It should be noted that, while the dynamic price fluctuates depending on the bids for a certain number of parking requests, it always ensures the collection of minimum parking fee.

## 7. Conclusions

Traffic management and accordingly convenient vehicle parking is a big challenge because of the booming number of vehicles worldwide. Therefore, a proper solution is required to mitigate the problem, ensure manageability, increase usability of parking slots and operate the traffic system informatively. In this paper, we presented the smart vehicle parking system architecture consisting of four layers: sensor nodes, edge gateway, LoRa gateway and Cloud. The sensor nodes are energy-efficient, secure, and collect multi-parametric data about the parking slots in near real-time. The edge layer processes sensor data before transmitting it to the cloud layer, and consequently to the end users. The LoRa gateway layer provides communication to ensure robust connection between the edge and cloud layers. We not only discussed the theoretical aspects of the smart parking systems but also provided a proof-of-concept with a specific realization. We demonstrated viability and usability of the proposed system for managing a parking area, and also discussed related services and design considerations. Even using off-the-self components, the sensor nodes achieve 20+ months of operation time which can be easily extended for a production ready sensor node. We took the latest communication technology LoRa along with nRF into use to effectively increase the energy-efficiency and coverage area. Furthermore, we presented a dynamic pricing algorithm for maximizing profit for the parking management authorities.

In future, we plan to implement AI-based computation in edge gateways for optimized management through cooperative decision making and running QoS services based on the vehicle parking data. This would facilitate better task allocation, enhance inter-gateway data synchronization and reduce network traffic by using AI-predicted parking usage patterns.

## Figures and Tables

**Table 1:** Comparison of earlier and related parking systems.

System/Author	Vehicle Sensing	Edge/Cloud Support	Connection Medium	Coverage Area	Estimated Cost	Operational Run-Time	System Scalability
P. Sadhukhan [12]	Camera, Ultrasonic	Edge	Wi-Fi, GSM	Large	High	Medium	Good
Aydin et al. [13]	Magnetic	No	Zigbee, GPRS	Medium	Low	Medium	Fair
Hainalkar et al. [14]	IR, RFID	No	-	Small	Low	Short	Poor
Grodi et al. [15]	Ultrasonic	No	Zigbee	Medium	Low	Long	Fair
Ramaswamy et al. [16]	Camera, Ultrasonic	Edge	Ethernet	Small	Medium	Medium	Poor
Kanteti et al. [18]	PIR, IP camera	Edge	Ethernet	Small	Medium	Medium	Poor
Teller et al. [19]	Camera	Edge	Ethernet	Medium	Medium	Medium	Poor
Proposed System	3D IMU, Water-level, Environment	Edge & Cloud	nRF, LoRa	Large	Low	Long	Good

**Table 2:** Example pricing schemes for our dynamic pricing algorithm.

Price	$N_{\mathrm{opt}}$	Value of *f* (€)
15	2	30
10	5	50
3	12	36

**Table 3:** LZW compression and decompression latency on edge and LoRa gateway, respectively, in different configuration options.

Data Set	Number of Nodes	Size [bytes]		Avg. Latency [ms]		Avg. Latency/Byte [μs]	
		Original	Compressed	Compress	Decompress	Compress	Decompress
Real-life	1	4	6	0.1755	0.0204	43.8695	5.0913
	10	40	47	0.1850	0.0366	4.6256	0.9139
	50	200	194	0.1591	0.0603	0.7959	0.3017
	100	400	395	0.3683	0.1568	0.9208	0.3920
True-random	1	4	194	0.1221	0.0143	30.5475	3.5720
	10	40	157	0.1610	0.0314	4.0252	0.7862
	50	200	395	0.2111	0.0801	1.0554	0.4005
	100	400	268	0.2431	0.1331	0.6078	0.3328

## Abbreviations

The following abbreviations are used in this manuscript:

AC	Alternating Current
ADC	Analog-Digital Converter
AES	Advanced Encryption Standard
AI	Artificial Intelligence
AVR	Alf-Egil Bogen Vegard Wollan RISC
BLE	Bluetooth Low Energy
CMOS	Complementary metal–oxide–semiconductor
CRC	Cyclic Redundancy Check
DC	Direct Current
DOF	Degree of Freedom
GPRS	General Packet Radio Service
GPS	Global Positioning System
GSM	Global System for Mobile
ID	Identity
IMU	Inertial Measurement Unit
IoT	Internet of Things
IP	Internet Protocol
IR	Infra-red
LCD	Liquid Crystal Display
LSB	Least Significant Bit
LTE	Long Term Evolution
LZW	Lempel-Ziv-Welch
MCU	Micro-controller Unit
NB	Narrow-band
PCB	Printed Circuit Board
PIR	Passive Infra-red
QoS	Quality of Service
RFID	Radio Frequency Identification
RGB	Red Green Blue
SFTP	Secure File Transfer Protocol
SMS	Short Message Service
UWB	Ultra-wideband
WSN	Wireless Sensor Network
